# A prediction model for neonatal mortality in low- and middle-income countries: an analysis of data from population surveillance sites in India, Nepal and Bangladesh

**DOI:** 10.1093/ije/dyy194

**Published:** 2018-10-15

**Authors:** Tanja A J Houweling, David van Klaveren, Sushmita Das, Kishwar Azad, Prasanta Tripathy, Dharma Manandhar, Melissa Neuman, Erik de Jonge, Jasper V Been, Ewout Steyerberg, Anthony Costello

**Affiliations:** 1 Department of Public Health, Erasmus MC University Medical Center Rotterdam, Rotterdam, The Netherlands; 2 Institute for Global Health, University College London, London, UK; 3 Department of Biomedical Data Sciences, Leiden University Medical Center, Leiden, The Netherlands; 4 Predictive Analytics and Comparative Effectiveness Center, Institute for Clinical Research and Health Policy Studies, Tufts Medical Center, Boston, USA; 5 Society for Nutrition, Education and Health Action (SNEHA), Mumbai, India; 6 Perinatal Care Project (PCP), Diabetic Association of Bangladesh (BADAS), Dhaka, Bangladesh; 7 Ekjut, West Singhbhum, India; 8 Mother and Infant Research Activities (MIRA), Kathmandu, Nepal; 9 Erasmus MC University Medical Center Rotterdam—Sophia Children’s Hospital, Rotterdam, The Netherlands

**Keywords:** neonatal mortality, prognostic model, Asia, demographic surveillance, pregnancy, delivery

## Abstract

**Background:**

In poor settings, where many births and neonatal deaths occur at home, prediction models of neonatal mortality in the general population can aid public-health policy-making. No such models are available in the international literature. We developed and validated a prediction model for neonatal mortality in the general population in India, Nepal and Bangladesh.

**Methods:**

Using data (49 632 live births, 1742 neonatal deaths) from rural and urban surveillance sites in South Asia, we developed regression models to predict the risk of neonatal death with characteristics known at (i) the start of pregnancy, (ii) start of delivery and (iii) 5 minutes post partum. We assessed the models’ discriminative ability by the area under the receiver operating characteristic curve (AUC), using cross-validation between sites.

**Results:**

At the start of pregnancy, predictive ability was moderate {AUC 0.59 [95% confidence interval (CI) 0.58–0.61]} and predictors of neonatal death were low maternal education and economic status, short birth interval, primigravida, and young and advanced maternal age. At the start of delivery, predictive ability was considerably better [AUC 0.73 (95% CI 0.70–0.76)] and prematurity and multiple pregnancy were strong predictors of death. At 5 minutes post partum, predictive ability was good [AUC: 0.85 (95% CI 0.80–0.89)]; very strong predictors were multiple birth, prematurity and a poor condition of the infant at 5 minutes.

**Conclusions:**

We developed good performing prediction models for neonatal mortality. Neonatal deaths are highly concentrated in a small group of high-risk infants, even in poor settings in South Asia. Risk assessment, as supported by our models, can be used as a basis for improving community- and facility-based newborn care and prevention strategies in poor settings.

**Key Messages**

- To our knowledge, this is the first prediction model of neonatal mortality in the general population in low- and middle-income countries published in an English-language international peer-reviewed journal.
- Using data on 49 632 live births and 1742 neonatal deaths from population surveillance sites in South Asia, we were able to develop good performing prediction models based on characteristics known at (i) the start of pregnancy, (ii) the start of delivery and (iii) 5 minutes post partum.
- Especially at 5 minutes post partum, predictive ability was high and strong predictors were multiple birth, prematurity and a poor condition of the infant.
- Risk assessment, as supported by our models, can be used as a basis for improving community- and facility-based newborn care and prevention strategies in poor settings.
- Our findings suggest that improved (community or facility-based) management of high-risk infants, combined with population-level strategies to reduce the prevalence of important risk factors, can substantially reduce neonatal mortality.

## Introduction

Worldwide, every year, nearly 3 million infants do not survive the first 28 days of life.^1^ Nearly all (99%) of these deaths occur in low- and middle-income countries.^2^ In poorer parts of India and Bangladesh, 35–65 babies in 1000 live births die in the neonatal period.^3^ For public-health policy-making and management of pregnancy, delivery and the newborn period, including proper risk selection and institution of selective care pathways for high-risk pregnancies, it is important to be able to predict which infants are at a high risk of neonatal death.

Prediction models of neonatal mortality are largely restricted to high-income countries, which account for only 1% of neonatal deaths. These models focus on infants in neonatal intensive-care units.^4–6^ Existing models for low- and middle-income countries are few and again focus on neonatal intensive-care patients.^7^ In poor settings, where many neonatal deaths occur at home,^8^ prediction models of neonatal mortality in the general population, rather than for selective high-risk patients only, can aid public-health policy-making and decision-making by family members and community health workers (e.g. through early recognition of potential problems). To our knowledge, no such models for neonatal mortality have been published in English-language international peer-reviewed journals.

Whereas prediction models for neonatal mortality are scarce, there is quite a good understanding of the causes of and risk factors for neonatal death in low- and middle-income countries. Preterm birth, neonatal infections and birth asphyxia account for around 80% of neonatal deaths.^1^^,^^2^ Direct risk factors include young and relatively advanced maternal age, maternal under-nutrition, primiparity and high parity, short pregnancy interval, multiple pregnancy, maternal health problems during pregnancy, malpresentation, problems during delivery, male infant sex (with exceptions in settings with strong son preference), low birth weight and exposure to infections.^2^^,^^9^^,^^10^ Low socio-economic position of the mother is an important underlying risk factor for neonatal death.^11^

The advantage of prediction models is that they formally combine risk factors, allowing more accurate risk estimation.^4^ Yet, as many births in poor settings occur at home without skilled care, good data on neonatal mortality and its risk factors remain scarce. Demographic surveillance sites in South Asia, in which the full population is followed up and all women were interviewed post partum, do provide such data, offering a unique opportunity to develop a prediction model for neonatal mortality in poor settings.

We aimed to develop and validate a prediction model for neonatal mortality in the general population in low- and middle-income countries, with specific reference to South Asia, using data from four surveillance sites.

## Methods

We used prospectively collected data from surveillance sites in rural Nepal (Makwanpur district, surveillance population of 170 000) and Bangladesh (Moulvibazar, Bogra and Faridpur district, 500 000) and rural (five districts in the states of Odisha and Jharkhand, 228 000) and urban (informal slum settlements in Mumbai, 283 000) India.^12–16^ The surveillance systems and data-collection tools were comparable across the sites. At each site, the full population (in the Nepal site, a closed cohort of women) in a geographically defined area was followed up on a continuous basis, and all births and birth outcomes were recorded. Local key informants, typically covering around 250 households, were responsible for reporting all births, birth outcomes and deaths to women of reproductive age to a salaried interviewer who met with the key informant on a monthly or fortnightly basis. The interviewer verified all reported events and paid the key informant a small financial incentive (more or less $1, depending on the site) for each correct identification. In the Nepal site, local female enumerators visited all cohort members in their area every month to record menstrual status. In each site, all women who had given birth, or a family member if the woman had died, were interviewed at around 6 weeks post partum, and detailed information about the mother and the pregnancy, delivery and newborn period was recorded. The questionnaires were similar across the sites, with some adaptations to the local context, e.g. in the way household assets were measured (see footnote to Table 1). The sites were set up for randomized–controlled trials of community-based interventions with participatory women’s groups. We only included data from the control arms of the trials. We included data from all South Asian sites of which the women’s group trial results have been published. The data were collected between 2001 and 2011 [Bangladesh 2005–11, Jharkhand/Odisha (India) 2005–09, Mumbai (India) 2006–09, Nepal 2001–03].

**Table 1.** Distribution of live births and neonatal deaths across risk factors, by study site

		Bangladesh		Jharkhand/Odisha, India		Mumbai, India		Nepal	
		# deliveries (%)	nnd	# deliveries (%)	nnd	# deliveries (%)	nnd	# deliveries (%)	nnd
Total per site		30 115	1041	8817	518	7478	64	3222	119
Time (years)	1	4923 (16.3)	199	2920 (33.1)	153	2643 (35.3)	22	1762 (54.7)	71
	2	5041 (16.7)	203	2972 (33.7)	177	2598 (34.7)	23	1460 (45.3)	48
	3	5234 (17.4)	175	2925 (33.2)	188	2237 (29.9)	19		
	4	4773 (15.8)	156						
	5	4204 (14.0)	133						
	6	5940 (19.7)	175						
Age	<18	986 (3.3)	40	214 (2.6)	25	58 (0.8)	1	53 (1.6)	4
	18–20	7591 (25.2)	306	1610 (19.4)	134	1273 (17.1)	11	361 (11.2)	13
	21–23	5799 (19.3)	195	1660 (20.0)	90	2006 (26.9)	12	733 (22.7)	23
	24–26	6406 (21.3)	164	1721 (20.8)	90	2005 (26.9)	15	556 (17.3)	20
	27–29	3556 (11.8)	117	1121 (13.5)	64	1069 (14.3)	13	448 (13.9)	13
	30–32	3030 (10.1)	109	1081 (13.0)	52	675 (9.0)	6	383 (11.9)	18
	33–35	1470 (4.9)	51	546 (6.6)	34	241 (3.2)	4	251 (7.8)	13
	>35	1271 (4.2)	58	337 (4.1)	15	138 (1.8)	2	437 (13.6)	15
	Missing	6	1	527	14	13	0		
Birth interval	Primigravida	10 090 (36.6)	372	2446 (28.2)	200	2367 (65.5)	17	609 (100.0)	23
(months)	<15	610 (2.2)	45	314 (3.6)	25	87 (2.4)	1		
	15–26	2699 (9.8)	89	1730 (20.0)	88	372 (10.3)	4		
	27–68	9731 (35.3)	279	3986 (46.0)	178	644 (17.8)	6		
	>68	4441 (16.1)	136	183 (2.1)	10	144 (4.0)	0		
	Missing	2544	120	158	17	3864	36	2613	96
Education^a^	No school	7107 (23.6)	320	5974 (67.8)	372	2094 (28.9)	26	2769 (86.0)	103
	Primary	10 076 (33.5)	369	448 (5.1)	26	397 (5.5)	5	302 (9.4)	9
	Secondary	12 582 (41.9)	345	2317 (26.3)	118	4037 (55.8)	29	146 (4.5)	6
	BSc/MSc	297 (1.0)	5	78 (0.9)	2	706 (9.8)	1	3 (0.1)	1
	Missing	53	2			244	3	2	0
Illiterate	No	21 516 (71.5)	654	2709 (30.7)	141	5328 (73.7)	42	710 (22.0)	25
	Yes	8585 (28.5)	386	6108 (69.3)	377	1906 (26.3)	19	2510 (78.0)	94
	Missing	14	1			244	3	2	0
Household wealth	Poorest	10 046 (33.4)	413	1565 (17.8)	110	1745 (23.3)	23	1792 (55.8)	73
(tertiles)^b^	Middle	10 839 (36.0)	369	3667 (41.6)	225	3534 (47.3)	27	1138 (35.4)	38
	Least poor	9228 (30.6)	259	3584 (40.7)	182	2199 (29.4)	14	283 (8.8)	8
	Missing	2	0	1	1			9	0
1 ANC visit	No	12 875 (42.8)	488	2541 (28.8)	169	2057 (27.5)	22	2676 (83.3)	98
	Yes	17 236 (57.2)	553	6273 (71.2)	349	5421 (72.5)	42	535 (16.7)	21
	Missing	4	0	3	0			11	0
4+ ANC visits	No	25 350 (84.2)	897	6784 (76.9)	419	2483 (33.2)	33	3079 (95.6)	113
	Yes	4764 (15.8)	144	2033 (23.1)	99	4995 (66.8)	31	141 (4.4)	6
	Missing	1	0					2	0
Tetanus vaccination	No	11 759 (39.0)	426	1485 (16.8)	110	474 (6.3)	13	215 (21.4)	8
	Yes	18 354 (61.0)	615	7332 (83.2)	408	7004 (93.7)	51	790 (78.6)	33
	Missing	2	0					2217	78
Premature	No	28 332 (94.7)	672	8290 (94.9)	382	7082 (95.0)	27	3140 (97.5)	84
	Yes	1600 (5.3)	362	445 (5.1)	130	374 (5.0)	15	82 (2.5)	35
	Missing	183	7	82	6	22	22		
Pregnancy complications	No	25 495 (84.7)	775	6860 (77.8)	372	7334 (98.9)	0		
	Yes	4588 (15.3)	265	1957 (22.2)	146	80 (1.1)	0		
	Missing	32	1			64	64	3222	119
Season^c^	Warm	7307 (24.3)	233	3106 (35.2)	157	1923 (25.7)	15	1407 (43.7)	44
	Rainy	12 106 (40.2)	416	2944 (33.4)	158	2380 (31.8)	20	1013 (31.4)	30
	Cold	10 702 (35.5)	392	2767 (31.4)	203	3175 (42.5)	29	802 (24.9)	45
Delivery location	Home	23 487 (78.6)	773	7031 (79.9)	428	952 (12.7)	17	3162 (98.1)	115
	Institutional	6403 (21.4)	260	1769 (20.1)	90	6526 (87.3)	47	60 (1.9)	4
	Missing	225	8	17	0				
Labour duration >24 h	No	23 994 (79.7)	770	7511 (85.2)	405	7271 (97.3)	57	2618 (81.3)	81
	Yes	6106 (20.3)	270	1305 (14.8)	113	202 (2.7)	2	604 (18.7)	38
	Missing	15	1	1	0	5	5		
Delivery complications	No	27 898 (92.9)	881	7252 (82.3)	393	7359 (98.4)	59	1782 (55.3)	51
	Yes	2123 (7.1)	157	1558 (17.7)	125	119 (1.6)	5	1439 (44.7)	68
	Missing	94	3	7	0			1	0
Presentation	Breech	559 (1.9)	75	96 (1.1)	29			18 (0.6)	3
	Normal	25 955 (86.8)	868	8509 (97.6)	475	54 (4.5)	54	3186 (99.4)	115
	Caesarean	3396 (11.4)	81	117 (1.3)	5	1136 (95.5)	9	2 (0.1)	0
	Missing	205	17	95	9	6288	1	16	1
Mother died	No	30 065 (99.8)	1034	8774 (99.5)	510	7475 (100.0)	63	3209 (99.6)	115
	Yes	50 (0.2)	7	43 (0.5)	8	3 (0.0)	1	13 (0.4)	4
Sex of baby	Male	15 536 (51.6)	615	4469 (50.7)	302	3939 (52.7)	38	1692 (52.5)	75
	Female	14 579 (48.4)	426	4348 (49.3)	216	3538 (47.3)	25	1530 (47.5)	44
	Missing					1	1		
Multiple birth	No	29 551 (98.1)	884	8613 (97.7)	449	7353 (98.3)	58	3162 (98.1)	110
	Yes	564 (1.9)	157	204 (2.3)	69	125 (1.7)	6	60 (1.9)	9
Size at birth	Small	5400 (17.9)	394	606 (6.9)	146	917 (12.4)	40	121 (3.8)	38
	Normal	22 119 (73.5)	509	8150 (92.4)	366	4311 (58.1)	7	3042 (94.4)	74
	Large	2595 (8.6)	138	61 (0.7)	6	2193 (29.6)	15	59 (1.8)	7
	Missing	1	0			57	2		
Looking abnormal	No	21 973 (92.4)	592	8422 (95.6)	437	7432 (99.4)	53	3149 (97.7)	95
	Yes	1810 (7.6)	254	392 (4.4)	80	46 (0.6)	11	73 (2.3)	24
	Missing	6332	195	3	1				
Breathed and cried	No	3977 (13.2)	402	41 (0.5)	8	117 (1.6)	19		
immediately	Yes	26 138 (86.8)	639	8776 (99.5)	510	7359 (98.4)	43		
	Missing					2	2	3222	119
Condition	Poor	1826 (6.1)	387	281 (3.2)	145				
at 5 min	Good	27 923 (93.9)	622	8441 (96.8)	351				
	Missing	366	32	95	22	7478	64	3222	119
Condition arms and legs	Normal	23 598 (99.1)	788	8677 (98.4)	438				
	Floppy	174 (0.7)	47	112 (1.3)	71				
	Stiff	39 (0.2)	10	28 (0.3)	9				
	Missing	6304	196			7478	64	3222	119

a Maternal education: ‘no schooling’ was used as reference category instead of BSc/MSc, because the latter group is extremely small.

b Household-wealth indicators included in the Principal Components Analysis were as follows: Bangladesh (electricity, radio/tape recorder, fan, television, telephone, generator, bicycle, fridge), Jharkhand/Odisha (India) (electricity, radio/tape recorder, fan, television, generator, bicycle, fridge), Mumbai (India) (electricity, radio/tape recorder, fan, television, telephone, bicycle, fridge). For Nepal, the wealth measure was based on predefined asset levels in the surveillance questionnaire, based on household ownership of one or more of the items on the list. These items were as follows: least poor (bus, truck, motorcycle, television, motor tractor, fridge, hand tractor, sewing machine/cassette player/fan/radio/camera/bicycle), middle (wall clock/iron), poorest (none of the above).

c Season was defined as follows: Bangladesh (rainy: June–October; cold: November–February; warm: March–May), Jharkhand/Odisha (India) (rainy: July–October; cold: November–February; warm: March–June), Mumbai (India) (rainy: June–September; cold: November–March; warm: October, April–May), Nepal (rainy: June–mid-September; cold: mid-November to mid-February; warm: mid-September to mid-November and mid-February to May).

Our outcome of interest was neonatal death, i.e. death in the first 28 days of life among live-born infants. All characteristics known to influence neonatal mortality as reported in the *Lancet Neonatal Survival* series,^2^^,^^17^ when available in our dataset, were included as predictors in our initial models. We also included season of birth—a predictor of neonatal death in at least one of our sites.^18^ All variables were based on the mother’s report or the report of a family member in the event of her death. Included characteristics at the start of pregnancy were: maternal age, maternal education (no school, primary, secondary, BSc/MSc) and literacy (can read, cannot read), household economic status (wealth tertiles, based on Principal Component Analysis)^19^ and pregnancy interval (using birth interval as proxy, categorized as <15, 15–26, 27–68, >68 months or primigravida).^10^ We included the following characteristics known at the start of delivery: at least one antenatal care (ANC) visit (y/n), at least four ANC visits (y/n), tetanus vaccination during pregnancy (y/n), premature birth (y/n, defined as gestational age of ≤8 months; gestational age in weeks not available), season of birth (warm-dry, rainy, cold) and pregnancy complications (y/n). Pregnancy complications were defined as any one of: reduced/no fetal movement, jaundice, fits/seizures/convulsions/lost consciousness. These complications were identified as the strongest independent predictors of neonatal mortality in a preliminary logistic regression analysis that also included: excessive vomiting, felt weak/tired, swollen feet/legs/face, severe stomach pain, looked pale, malaria, severe headache/dizziness/fainting, breathless when doing household tasks, blurred vision/spots before eyes, anaemia. Multiple birth (y/n) may or may not have been known at the start of delivery, depending on the quality of the ANC. The following characteristics known 5 minutes post partum were included: presentation/mode of delivery [normal, breech, caesarean section (C-section)], place of delivery (home, facility), labour duration (≤ or >24 hrs), delivery complications (y/n), maternal death (y/n), sex of baby, size of baby at birth (small, normal, large), looking abnormal (y/n), breathing/crying immediately after birth (y/n), condition of arms and legs of baby after birth (normal, floppy, stiff) and condition of baby at 5 minutes (‘crying well, breathing well, pink and active’, ‘poor or no cry, poor breathing, blue limbs or body, poorly active/no movement’). Delivery complications were defined as any one of the following: fever within 3 days prior to labour, retained placenta and haemorrhage (‘vaginal bleeding so much that you thought you were going to die’). Looking abnormal was mostly based on the question: ‘How did the baby look at birth, normal/abnormal?’

Most predictors were available for over 90% of deliveries (Table 1). Some variables were not available or had many missing values for the Mumbai (India) site (presentation; condition at 5 minutes; condition arms and legs) and rural Nepal (birth interval; tetanus vaccination; pregnancy complications; breathed and cried immediately; condition at 5 minutes; condition arms and legs). Because each variable was available for a considerable number of births, we used an advanced multiple imputation of missing values strategy (method of chained equations) to make efficient use of the available data.^20^ To maximize the use of all the available information, we included all potential predictors of neonatal mortality, as well as the site and the outcome, in the model for imputation of missing values. We used the R package ‘mice’ for multiple imputation.^21^ We developed three logistic regression models to predict the risk of death in the first 28 days of life at the individual level, based on characteristics known at (i) the start of pregnancy, (ii) the start of delivery and (iii) 5 minutes post partum.

We modelled possible non-linearity of the association between mother’s age and the risk of neonatal death with restricted cubic splines.^22^ We expressed the strength of the association between predictors and neonatal death by crude and adjusted odds ratios. We evaluated the contribution of each predictor by the difference in Akaike’s information criterion (ΔAIC) between multivariable models with and without the predictive factor, balancing the improvement in goodness of fit of a model with its increased complexity.^22^ We deleted variables with negligible predictive contribution, i.e. when the χ^2^ test statistic minus twice the degrees of freedom was relatively small (below 10).

We assessed the discriminative ability of each model by the area under the curve (AUC) of the receiver operating characteristic (ROC) curve. The AUC can be interpreted as the probability that the risk prediction of a randomly chosen neonatal death is higher than the risk prediction of a randomly chosen neonatal survivor. We determined the AUC of the models within each of the four sites (‘apparent AUC’). We also used a cross-validation approach between sites to obtain a more realistic presentation of the AUC in independent settings (‘cross-validated AUC’). Cross-validation means that the model is consecutively fitted in three of the four sites and validated—with the AUC—in the site that was left out when fitting the model. To obtain overall AUCs—both apparent and cross-validated—we used random-effects meta-analyses of the four site-specific AUCs.^23^

For calculation of an individual’s probability of neonatal death, we present the prediction models with nomograms.^22^^,^^24^ For regression analysis and construction of nomograms, we used the R package ‘rms’.^21^

Approval for the trials of which we used the data for our secondary analysis was received from the Research Ethics Committee at the UCL Institute of Child Health and appropriate national Ethics Committees.^13–16^

## Results

Across the sites, 1742 neonatal deaths occurred in 49 632 live births, with the neonatal mortality rate (NMR) varying from 58.8/1000 in rural Jharkhand/Odisha (India) to 36.9/1000 in Nepal, 34.6/1000 in Bangladesh and 8.6/1000 in informal settlements in Mumbai (India) (Table 1).

The following characteristics were very strongly associated with neonatal death [univariable odds ratios (ORs), Supplementary Table 1, available as Supplementary data at *IJE* online]: breech delivery, premature birth, mother died, multiple birth, small size at birth, looking abnormal, not immediately crying or breathing, poor condition at 5 minutes, and infant had floppy or stiff arms and legs. The other included characteristics were also associated, though less strongly, with neonatal death in most sites.

Table 2 presents the prediction models. At the start of pregnancy, a high educational attainment was associated with a lower odds of death and low economic status was associated with a higher odds of death (Table 2; Supplementary Table 2, available as Supplementary data at *IJE* online). Also, a very short birth interval and births to primigravid, younger (especially <18 years) and older (35+) women were associated with a higher odds of death. Socio-economic (ΔAIC education: 35; economic status: 12) and demographic characteristics (ΔAIC birth interval: 31; maternal age: 14) were equally strong predictors of neonatal death. At the start of pregnancy, the predictive ability of the model was moderate {apparent AUC: 0.59 [95% confidence interval (CI) 0.58–0.61]; cross-validated AUC 0.58 [95% CI 0.56–0.59]}.

**Table 2.** Multivariable associations between neonatal mortality and risk factors at start of pregnancy, start of delivery and 5 minutes after birth

		Start of pregnancy	Start of delivery	After birth	Start of delivery (including multiple birth)
Site		**198**	**166**	**147**	**174**
	Bangladesh	1	1	1	1
	Jharkhand/Odisha, India	1.66 (1.46, 1.89)	1.61 (1.40, 1.84)	2.04 (1.76, 2.37)	1.62 (1.41, 1.86)
	Mumbai, India	0.27 (0.20, 0.34)	0.27 (0.21, 0.35)	0.37 (0.28, 0.50)	0.25 (0.19, 0.33)
	Nepal	0.81 (0.65, 1.00)	0.95 (0.76, 1.18)	1.27 (1.00, 1.61)	0.97 (0.78, 1.21)
Age		**14**			
	<18	1.55 (1.25, 1.92)			
	18–20	1.30 (1.14, 1.48)			
	21–23	1.11 (1.05, 1.17)			
	24–26	1			
	27–29	0.99 (0.96, 1.03)			
	30–32	1.05 (0.98, 1.12)			
	33–35	1.14 (1.00, 1.29)			
	>35	1.27 (1.04, 1.54)			
Birth interval (months)		**31**	**41**	**20**	**55**
	Primigravida	1.34 (1.14, 1.57)	1.40 (1.22, 1.60)	1.25 (1.08, 1.44)	1.50 (1.31, 1.72)
	<15	2.18 (1.69, 2.82)	1.90 (1.48, 2.45)	1.82 (1.38, 2.40)	1.98 (1.53, 2.58)
	15–26	1.11 (0.93, 1.33)	1.01 (0.83, 1.22)	1.01 (0.82, 1.23)	1.04 (0.85, 1.26)
	27–68	1	1	1	1
	>68	1.16 (0.95, 1.41)	1.07 (0.88, 1.30)	1.02 (0.83, 1.25)	1.04 (0.85, 1.26)
Education		**35**	**50**	**41**	**49**
	No school	1	1	1	1
	Primary	0.84 (0.73, 0.97)	0.79 (0.68, 0.91)	0.80 (0.69, 0.94)	0.80 (0.69, 0.93)
	Secondary	0.65 (0.57, 0.75)	0.60 (0.52, 0.69)	0.62 (0.53, 0.71)	0.61 (0.53, 0.70)
	BSc/MSc	0.39 (0.20, 0.76)	0.30 (0.15, 0.59)	0.37 (0.18, 0.75)	0.25 (0.12, 0.50)
Household wealth (tertiles)		**12**	**12**		**13**
	1	1.31 (1.14, 1.50)	1.32 (1.15, 1.52)		1.33 (1.16, 1.53)
	2	1.12 (0.99, 1.27)	1.10 (0.97, 1.25)		1.11 (0.97, 1.26)
	3	1	1		1
Premature			**1658**	**745**	**1372**
	No		1	1	1
	Yes		11.11 (9.89, 12.47)	7.62 (6.59, 8.82)	9.65 (8.56, 10.88)
Pregnancy complications			**46**	**22**	**40**
	No		1	1	1
	Yes		1.55 (1.37, 1.75)	1.40 (1.22, 1.59)	1.51 (1.33, 1.71)
Season			**13**	**23**	**15**
	Warm		1	1	1
	Rainy		1.00 (0.88, 1.14)	1.05 (0.91, 1.21)	1.01 (0.89, 1.16)
	Cold		1.24 (1.09, 1.41)	1.38 (1.20, 1.59)	1.27 (1.11, 1.44)
Presentation				**49**	
	Caesarean			0.47 (0.36, 0.60)	
	Breech			1.75 (1.33, 2.32)	
	Normal			1	
Sex baby				**31**	
	Male			1.38 (1.24, 1.54)	
	Female			1	
Multiple birth				**333**	**508**
	No			1	1
	Yes			6.78 (5.52, 8.32)	7.67 (6.43, 9.16)
Size at birth				**82**	
	Small			1.50 (1.31, 1.73)	
	Normal			1	
	Large			2.29 (1.89, 2.77)	
Condition at 5 min				**1110**	
	Poor			10.09 (8.81, 11.56)	
	Good			1	
Condition arms				**119**	
	Normal			1	
	Floppy			5.25 (3.91, 7.05)	
AUC apparent validation					
Bangladesh		0.59 (0.58, 0.61)	0.73 (0.71, 0.75)	0.83 (0.81, 0.84)	0.75 (0.74, 0.77)
Jharkhand/Odisha, India		0.60 (0.57, 0.62)	0.68 (0.65, 0.71)	0.80 (0.78, 0.82)	0.71 (0.68, 0.73)
Mumbai India		0.63 (0.56, 0.70)	0.75 (0.68, 0.83)	0.92 (0.88, 0.96)	0.75 (0.68, 0.82)
Nepal		0.54 (0.47, 0.61)	0.71 (0.66, 0.77)	0.84 (0.79, 0.89)	0.73 (0.67, 0.79)
Pooled average		0.59 (0.58, 0.61)	0.72 (0.68, 0.75)	0.85 (0.80, 0.89)	0.73 (0.70, 0.76)
AUC cross-validation					
Bangladesh		0.58 (0.56, 0.60)	0.72 (0.70, 0.74)	0.81 (0.79, 0.83)	0.74 (0.72, 0.76)
Jharkhand/Odisha, India		0.58 (0.56, 0.61)	0.67 (0.65, 0.70)	0.79 (0.77, 0.82)	0.70 (0.67, 0.73)
Mumbai, India		0.62 (0.55, 0.69)	0.75 (0.68, 0.83)	0.90 (0.85, 0.95)	0.75 (0.67, 0.82)
Nepal		0.53 (0.47, 0.60)	0.71 (0.65, 0.77)	0.84 (0.79, 0.89)	0.72 (0.66, 0.78)
Pooled average		0.58 (0.56, 0.59)	0.71 (0.68, 0.74)	0.83 (0.79, 0.86)	0.73 (0.70, 0.75)

ΔAIC is reported behind the predictors in bold font; odd ratios (95% confidence intervals) are reported behind predictor levels in regular font.

At the start of delivery, prematurity was a very strong predictor of neonatal death [ΔAIC: 1658; OR 11.11 (95% CI 9.89–12.47)]. Less strong, but still predictive, were health problems during pregnancy and delivery in the cold season. Low maternal socio-economic position and short birth interval were also important predictors. Predictive ability at the start of delivery was considerably better than at the start of pregnancy [AUC: 0.72 (95% CI 0.68–0.75)]. Multiple pregnancy was a strong predictor of neonatal death [ΔAIC: 508; OR 7.67 (95% CI 6.43–9.16)]. When information about multiple pregnancy was available at the start of delivery, the predictive ability improved [apparent AUC: 0.73 (95% CI 0.70–0.76); cross-validated AUC 0.73 (95% CI 0.70–0.75)].

At 5 minutes post partum, prematurity [ΔAIC: 745; OR 7.62 (6.59–8.82)], a poor condition of the infant [ΔAIC: 1110; OR 10.09 (95% CI 8.81–11.56)] and multiple birth [ΔAIC: 333; OR 6.78 (95% CI 5.52–8.32)] were highly predictive of neonatal death. Less predictive, but still important, were low maternal education, short birth interval, floppy or stiff arms and legs of the baby, small or large infant size at birth, breech delivery, male infant, health problems during pregnancy and delivery in the cold season. The predictive ability of this model was high [apparent AUC: 0.85 (95% CI 0.80–0.89); cross-validated AUC 0.83 (95% CI 0.79–0.86)]. A substantial proportion of deaths was associated with the three risk factors with the highest ΔAIC at time of delivery (60.1% of deaths and 9.3% of births had any one of these risk factors).

**Figure 1.**
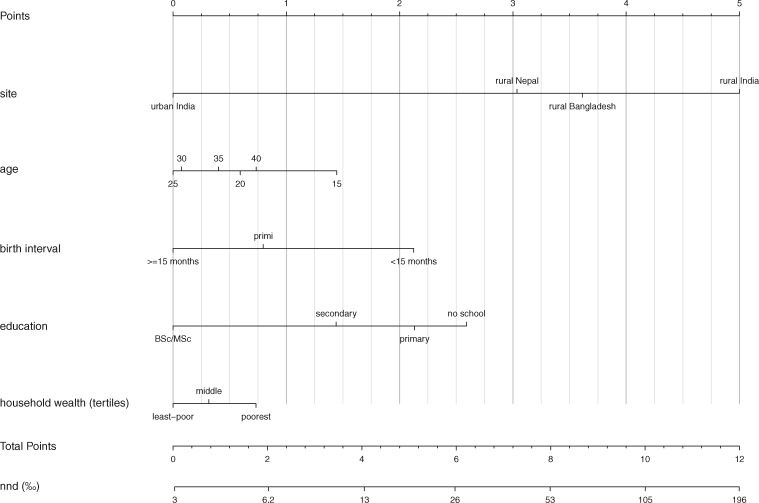
Nomogram of the prediction of neonatal mortality at start of pregnancy. To estimate an infant’s probability of neonatal death, first determine all of its risk-factor characteristics [educational attainment of its mother, (estimated) birth interval, etc.]. Second, read the risk points associated with each risk factor by drawing a line up from the predictor value to the ‘Points’ axis. Third, add up the points for all risk factors to obtain the total points for that infant. The probability of neonatal death can be read by moving vertically from the ‘Total Points’ axis to the ‘nnd’ axis. The predictor ‘site’ can be used to take regional differences in NMR into account. When using the nomograms outside of our study populations, readers are advised to use the site with an NMR closest to their own study population.

**Figure 2.**
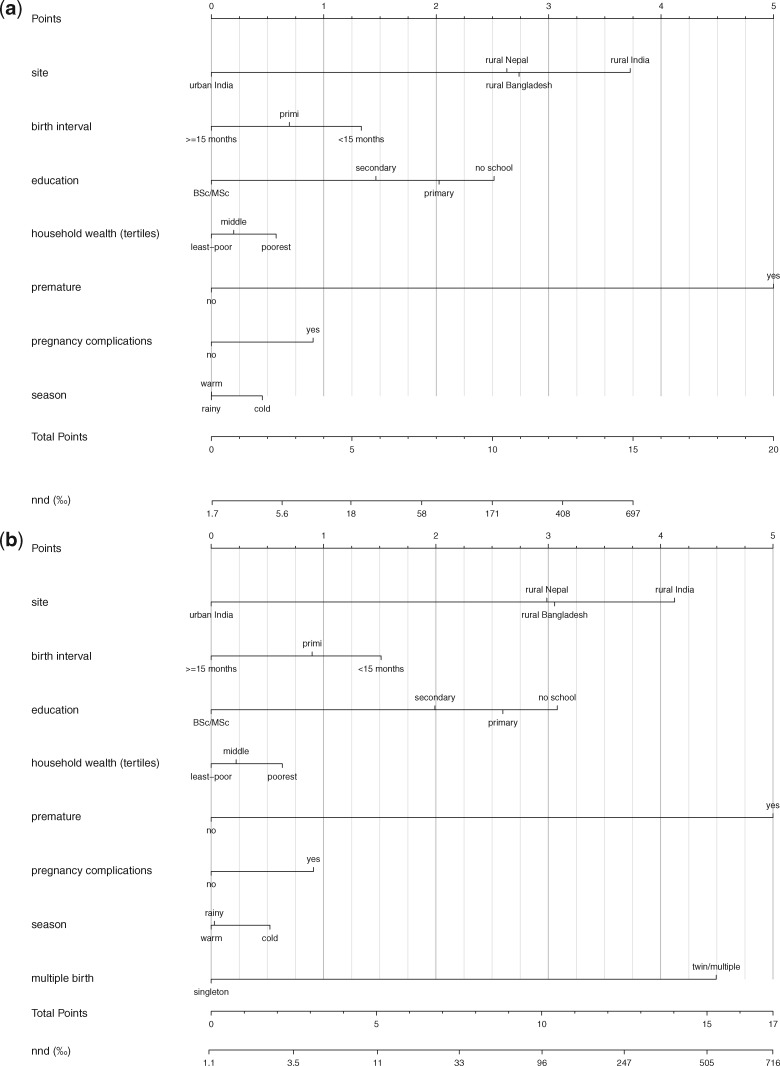
(**A**) Nomogram of the prediction of neonatal mortality at the start of delivery (without information on singleton/multiple pregnancy). (**B**) Nomogram of the prediction of neonatal mortality at the start of delivery (with information on singleton/multiple pregnancy).

**Figure 3.**
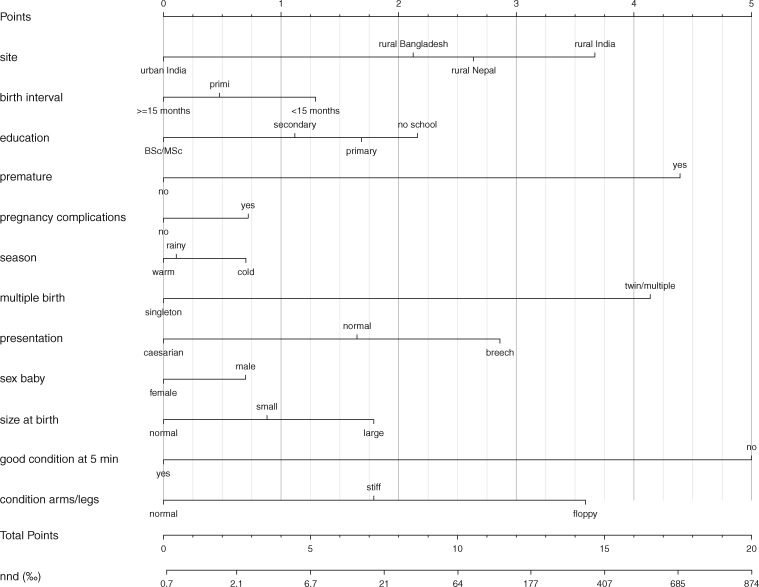
Nomogram of the prediction of neonatal mortality at 5 minutes after delivery.

The prognostic nomograms corresponding to the three models are presented in the nomograms of Figures 1–3 (see explanation underneath Figure 1; nomogram details are in Supplementary Table 3, available as Supplementary data at *IJE* online). Using Figure 3, e.g. a singleton male infant (0.7 points), with a small size at birth (0.9 points), who presented normally (1.7 points), but was born prematurely (4.4 points) in the cold season (0.7 points) in Jharkhand/Odisha (India) (3.7 points), to a primigravid (0.5 points) mother with no schooling (2.2 points) had an estimated mortality risk of 384/1000 if the infant was in good condition at 5 minutes, with arms/legs in normal condition. If the same infant was in a poor condition at 5 minutes (5 points), but with arms/legs in normal condition, the mortality risk amounted to 863/1000.

## Discussion

We developed and validated prognostic models for neonatal mortality in the general population in low- and middle-income countries, with specific reference to South Asia, on the basis of risk factors known at (i) the start of pregnancy, (ii) the start of delivery and (iii) 5 minutes post partum. At the start of pregnancy, prediction of neonatal death was difficult, although infants born to women of lower socio-economic position and to women with certain demographic characteristics (young or advanced age, very short birth interval, primigravida) were at a higher risk of neonatal death. Predictive ability improved at the start of delivery, where multiple pregnancy and a premature start of delivery were highly predictive of neonatal death. Predictive ability was high at 5 minutes post partum, where prematurity, multiple birth and a poor condition of the infant were strong predictors of death. The models can be used to inform population-based prevention and more narrowly targeted interventions for high-risk infants.

### Methodological issues

Our models are based on large datasets from sites in which the full population was prospectively followed up and detailed information on predictors of neonatal death was collected, allowing precise prediction. Yet, recall bias is a potential problem, as information was based on the mother’s report at approximately 6 weeks post partum. Whereas we reduced this problem by using broad categories for variables such as size at birth, random error may remain substantial for such variables. Furthermore, the mother’s report may have been biased by the outcome (death/survival), with worse conditions reported for neonatal deaths, leading to inflated ORs for characteristics that mothers associate with death (e.g. infant condition at 5 minutes). Yet, for other predictors, such as multiple birth, such recall bias is probably minimal. Finally, whereas the high number of missing values in some predictors in particular sites may be considered a limitation, we were able to develop our models based on multiple imputation of missing values using the substantial amount of available data. Nevertheless, this may have led to an under-estimation of the discriminative ability of the models. Despite these problems, we arguably used some of the best data available for general populations in poor settings (i.e. prospectively collected data from some of the largest networks of linked demographic surveillance sites in South Asia) where home births without skilled care are common and reliable vital registration systems are non-existent.

Our models are arguably generalizable to rural and poor urban South Asia. Our study sites ranged from informal settlements in megacity Mumbai, with a comparatively low NMR, to tribal areas in some of the poorest states in India, with a high NMR. The discriminative ability of the models—measured by the apparent and cross-validated AUC—was stable across sites, implying that the models are generally applicable across our study population. Our models are possibly less applicable to the top layer of South Asian society with a different cause-of-death pattern. Furthermore, their wider generalizability to other world regions needs further examination.

### Comparison with the literature and implications

To our knowledge, our study is the first to formally combine known risk factors for neonatal mortality into a prediction model for the general population in low- and middle-income countries. We developed models for three time points, i.e. onset of pregnancy, onset of delivery, immediately after birth—something we rarely encountered in the literature.

We found that three risk factors—preterm birth, multiple birth and poor condition at 5 minutes post partum—were associated with a very high risk of neonatal death. A substantial proportion of deaths was associated with these risk factors. Secondary prevention (improving outcomes among infants with these risk factors, rather than reducing risk-factor prevalence) can play an important role in preventing these deaths. Facility-based interventions to improve management of high-risk infants exist for poor settings.^25^^,^^26^ Whereas timely access to skilled care can be critical, it is often problematic in poor rural areas. Health-system strengthening to improve the quality and availability of care and demand-side interventions (e.g. conditional cash transfers) to reduce care-seeking delays are therefore important. Interventions also exist for community settings, including participatory women’s groups and home-based neonatal care by village health workers.^26^^,^^27^ Community-based management requires that care-givers are aware of important risk factors and react pro-actively to danger signs.^28^ This means anticipating potential problems in women with a multiple pregnancy and/or premature start of delivery where there is still time to travel to a facility, and early recognition and home management of problems among preterm infants and babies in a poor condition (e.g. bag-and-mask ventilation, kangaroo care, delayed bathing).^29^^,^^30^ Raising awareness about the importance of the above risk factors within community-based interventions and empowering families and communities to address these problems are therefore recommended. Similarly, these strategies can be used for the other described risk factors, including breech delivery (timely recognition and care-seeking) and delivery in the cold season (thermal care). Also, whereas infants are at the highest risk of death on the day of birth,^1^ these strategies are equally important for the late neonatal period (comprising 20–50% of deaths in our sites). So, rather than being competing strategies, population-level interventions to raise awareness and empower communities to act are a prerequisite for effective secondary prevention in settings where home births without professional care are common.

Combining the above strategies with population-level primary prevention to reduce the incidence of risk factors, e.g. by improving maternal nutrition, reducing indoor pollution and increased use of family planning, will help to further reduce neonatal mortality.^1^^,^^31^ Similarly, measures to improve living conditions and hygienic practices are important. Forty per cent of deaths in our sites occurred among infants without the three main risk factors; infections may have played an important role in these deaths, as well as in the death of high-risk infants.^1^

## Conclusions

We developed good performing prediction models for neonatal mortality in the general population in South Asia. We conclude that neonatal deaths are highly concentrated in a small group of high-risk infants, even in poor settings in South Asia. These high-risk infants can be identified based on characteristics available before or shortly after birth. Our models suggest that improved management of high-risk infants can substantially reduce neonatal mortality. Where health systems are weak, a high-risk approach should arguably include population-level strategies to raise awareness about important risk factors and empower community-based care-givers to take action. This should arguably be complemented with health-system strengthening to improve the uptake of facility-based care and quality of maternity and newborn care and action on the social determinants of health to reduce mortality in low-risk, as well as high-risk, infants.

## Funding 

This work was supported by the Economic and Social Research Council and the Department for International Development (grant number ES/I033572/1) and a Wellcome Trust Strategic Award (award number: 085417MA/Z/08/Z). T.A.J.H. was supported by an Erasmus University Rotterdam Research Excellence Initiative grant. D.v.K. was supported by the Netherlands Organization for Scientific Research (grant number 917.11.383). J.V.B. was supported by fellowship grants from the Netherlands Lung Foundation and the Erasmus University Medical Center. The funders had no role in study design, data collection and analysis, decision to publish or preparation of the manuscript. T.A.J.H. and D.v.K. had full access to all the data in the study and take responsibility for the integrity of the data and the accuracy of the data analysis.

**Conflict of interest:** None declared 

## Supplementary Material

Supplementary Tables

## References

[1] Lawn JE, Blencowe H, Oza S et al Every Newborn: progress, priorities, and potential beyond survival. Lancet 2014;384:189–205. 24853593 10.1016/S0140-6736(14)60496-7

[2] Lawn JE, Cousens S, Zupan J. 4 million neonatal deaths: when? Where? Why? Lancet 2005;365:891–900. 15752534 10.1016/S0140-6736(05)71048-5

[3] Houweling TA, Looman CW, Azad K et al The equity impact of community women’s groups to reduce neonatal mortality: a meta-analysis of four cluster randomised trials. Int J Epidemiol 2019;48:168–82. 10.1093/ije/dyx160 PMC6380297 29024995

[4] Schuit E, Hukkelhoven CW, Manktelow BN et al Prognostic models for stillbirth and neonatal death in very preterm birth: a validation study. Pediatrics 2012;129:e120–27. 22157141 10.1542/peds.2011-0803

[5] Draper ES, Manktelow B, Field DJ, James D. Prediction of survival for preterm births by weight and gestational age: retrospective population based study. BMJ 1999;319:1093–97. 10531097 10.1136/bmj.319.7217.1093 PMC28258

[6] Medlock S, Ravelli AC, Tamminga P, Mol BW, Abu-Hanna A. Prediction of mortality in very premature infants: a systematic review of prediction models. PLoS One 2011;6:e23441. 21931598 10.1371/journal.pone.0023441 PMC3169543

[7] Li L, Yu J, Wang J et al A prediction score model for risk factors of mortality in neonate with pulmonary hemorrhage: the experience of single neonatal intensive care unit in Southwest China. Pediatr Pulmonol 2008;43:997–1003. 18785623 10.1002/ppul.20897

[8] Houweling TA, Ronsmans C, Campbell OM, Kunst AE. Huge poor-rich inequalities in maternity care: an international comparative study of maternity and child care in developing countries. Bull World Health Organ 2007;85:745–54. 18038055 10.2471/BLT.06.038588 PMC2636501

[9] Dhaded SM, Somannavar MS, Vernekar SS et al Neonatal mortality and coverage of essential newborn interventions 2010–2013: a prospective, population-based study from low-middle income countries. Reprod Health 2015;12:S6. 10.1186/1742-4755-12-S2-S6 PMC4464215 26063125

[10] Conde-Agudelo A, Rosas-Bermudez A, Kafury-Goeta AC. Birth spacing and risk of adverse perinatal outcomes: a meta-analysis. JAMA 2006;295:1809–23. 16622143 10.1001/jama.295.15.1809

[11] Houweling TA, Kunst AE. Socio-economic inequalities in childhood mortality in low and middle income countries: a review of the international evidence. Br Med Bull 2010;93:7–26. 20007188 10.1093/bmb/ldp048

[12] Barnett S, Nair N, Tripathy P, Borghi J, Rath S, Costello AA. Prospective key informant surveillance system to measure maternal mortality—findings from indigenous populations in Jharkhand and Orissa, India. BMC Pregnancy Childbirth 2008;8:6. 18307796 10.1186/1471-2393-8-6 PMC2268911

[13] Tripathy P, Nair N, Barnett S et al Effect of a participatory intervention with women’s groups on birth outcomes and maternal depression in Jharkhand and Orissa, India: a cluster-randomised controlled trial. Lancet 2010;375:1182–92. 20207411 10.1016/S0140-6736(09)62042-0

[14] Manandhar DS, Osrin D, Shrestha BP et al Effect of a participatory intervention with women’s groups on birth outcomes in Nepal: cluster-randomised controlled trial. Lancet 2004;364:970–79. 15364188 10.1016/S0140-6736(04)17021-9

[15] More NS, Bapat U, Das S et al Community mobilization in Mumbai slums to improve perinatal care and outcomes: a cluster randomized controlled trial. PLoS Med 2012;9:e1001257. 22802737 10.1371/journal.pmed.1001257 PMC3389036

[16] Fottrell E, Azad K, Kuddus A et al The effect of increased coverage of participatory women’s groups on neonatal mortality in Bangladesh: a cluster randomized trial. JAMA Pediatr 2013;167:816–25. 23689475 10.1001/jamapediatrics.2013.2534 PMC5082727

[17] Darmstadt GL, Bhutta ZA, Cousens S, Adam T, Walker N, de Bernis L. Evidence-based, cost-effective interventions: how many newborn babies can we save? Lancet 2005;365:977–88. 15767001 10.1016/S0140-6736(05)71088-6

[18] Roy SS, Mahapatra R, Rath S et al Improved neonatal survival after participatory learning and action with women’s groups: a prospective study in rural eastern India. Bull World Health Organ 2013;91:426–33B. 24052679 10.2471/BLT.12.105171 PMC3777144

[19] Filmer D, Pritchett LH. Estimating wealth effects without expenditure data—or tears: an application to educational enrolments in states of India. Demography 2001;38:115–32. 11227840 10.1353/dem.2001.0003

[20] Van Buuren S, Groothuis-Oudshoorn K. MICE: multivariate imputation by chained equations. J Stat Soft 2011;45:1–67.

[21] R Core Team. *R: A Language and Environment for Statistical Computing, Version 2.15.*3 [computer program]. Vienna: R Foundation for Statistical Computing, 2013.

[22] Harrell F. Regression Modeling Strategies: With Applications to Linear Models, Logistic Regression, and Survival Analysis. New York: Springer, 2001.

[23] van Klaveren D, Steyerberg EW, Perel P, Vergouwe Y. Assessing discriminative ability of risk models in clustered data. BMC Med Res Methodol 2014;14:5. 24423445 10.1186/1471-2288-14-5 PMC3897966

[24] Steyerberg E. Clinical Prediction Models: A Practical Approach to Development, Validation, and Updating. New York: Springer, 2009.

[25] Lee AC, Katz J, Blencowe H et al National and regional estimates of term and preterm babies born small for gestational age in 138 low-income and middle-income countries in 2010. Lancet Glob Health 2013;1:e26–36. 25103583 10.1016/S2214-109X(13)70006-8 PMC4221634

[26] March of Dimes, PMNCH, Save the Children, WHO. Born Too Soon: The Global Action Report on Preterm Birth*.* Geneva: World Health Organization, 2012.

[27] Houweling TA, Tripathy P, Nair N et al The equity impact of participatory women’s groups to reduce neonatal mortality in India: secondary analysis of a cluster-randomised trial. Int J Epidemiol 2013;42:520–32. 23509239 10.1093/ije/dyt012 PMC3619953

[28] Mesko N, Osrin D, Tamang S et al Care for perinatal illness in rural Nepal: a descriptive study with cross-sectional and qualitative components. BMC Int Health Hum Rights 2003;3:3. 12932300 10.1186/1472-698X-3-3 PMC194728

[29] Wall SN, Lee AC, Niermeyer S et al Neonatal resuscitation in low-resource settings: what, who, and how to overcome challenges to scale up? Int J Gynaecol Obstet 2009;107(Suppl 1):S47–62, S63–44. 19815203 10.1016/j.ijgo.2009.07.013 PMC2875104

[30] Lawn JE, Mwansa-Kambafwile J, Barros FC, Horta BL, Cousens S. ‘Kangaroo mother care’ to prevent neonatal deaths due to pre-term birth complications. Int J Epidemiol 2011;40:525–28. 21062786 10.1093/ije/dyq172 PMC3066426

[31] Ramakrishnan U, Grant FK, Goldenberg T, Bui V, Imdad A, Bhutta ZA. Effect of multiple micronutrient supplementation on pregnancy and infant outcomes: a systematic review. Paediatr Perinat Epidemiol 2012;26:153–67. 22742608 10.1111/j.1365-3016.2012.01276.x

