# Supplementary material for: A prediction model for neonatal mortality in low- and middle-income countries: an analysis of data from population surveillance sites in India, Nepal and Bangladesh
Source: Int J Epidemiol. 2018 Oct 15;48(1):186–98. doi: 10.1093/ije/dyy194 (PMC6380321; doi:10.1093/ije/dyy194)
Supplement: Supplementary Tables [file dyy194_supplementary_tables.docx]

**Supplementary Table 1. Univariable Odds Ratios (95% confidence interval) for all Risk Factors, by Study Site**

|  |  | **Rural Bangladesh** | **Jharkhand/Odisha, India** | **Mumbai, India** | **Rural Nepal** |
| --- | --- | --- | --- | --- | --- |
|  |  |  |  |  |  |
| time (years) | 1 | 1 | 1 | 1 | 1 |
|  | 2 | 1.00 (0.82,1.22) | 1.15 (0.92,1.43) | 1.06 (0.59,1.91) | 0.81 (0.56,1.18) |
|  | 3 | 0.82 (0.67,1.01) | 1.24 (1.00,1.55) | 1.02 (0.55,1.89) |  |
|  | 4 | 0.80 (0.65,0.99) |  |  |  |
|  | 5 | 0.78 (0.62,0.97) |  |  |  |
|  | 6 | 0.72 (0.59,0.89) |  |  |  |
|  |  |  |  |  |  |
| age | <18 | 1.61 (1.13,2.29) | 2.40 (1.50,3.83) | 2.33 (0.30,17.92) | 2.19 (0.72,6.66) |
|  | 18-20 | 1.60 (1.32,1.94) | 1.65 (1.25,2.17) | 1.16 (0.53,2.53) | 1.00 (0.49,2.04) |
|  | 21-23 | 1.32 (1.07,1.64) | 1.04 (0.77,1.40) | 0.80 (0.37,1.71) | 0.87 (0.47,1.60) |
|  | 24-26 | 1 | 1 | 1 | 1 |
|  | 27-29 | 1.29 (1.02,1.65) | 1.10 (0.79,1.53) | 1.63 (0.77,3.45) | 0.80 (0.39,1.63) |
|  | 30-32 | 1.42 (1.11,1.82) | 0.92 (0.65,1.30) | 1.19 (0.46,3.08) | 1.32 (0.69,2.53) |
|  | 33-35 | 1.37 (0.99,1.88) | 1.20 (0.80,1.81) | 2.24 (0.74,6.80) | 1.46 (0.72,2.99) |
|  | >35 | 1.82 (1.34,2.47) | 0.84 (0.48,1.48) | 1.95 (0.44,8.62) | 0.95 (0.48,1.88) |
|  | missing | 7.61 (0.88,65.52) | 0.49 (0.28,0.88) |  |  |
|  |  |  |  |  |  |
| birth interval | primi gravida | 1.30 (1.11,1.52) | 1.91 (1.55,2.35) | 0.77 (0.30,1.96) |  |
| (months) | <15 | 2.70 (1.95,3.74) | 1.85 (1.20,2.86) | 1.24 (0.15,10.39) |  |
|  | 15-26 | 1.16 (0.91,1.47) | 1.15 (0.88,1.49) | 1.16 (0.32,4.12) |  |
|  | 27-68 | 1 | 1 | 1 |  |
|  | >68 | 1.07 (0.87,1.32) | 1.24 (0.64,2.38) |  |  |
|  | missing | 1.68 (1.35,2.09) | 2.58 (1.53,4.36) | 1.00 (0.42,2.38) |  |
|  |  |  |  |  |  |
| education | no school | 1 | 1 | 1 | 1 |
|  | primary | 0.81 (0.69,0.94) | 0.93 (0.62,1.40) | 1.01 (0.39,2.66) | 0.80 (0.40,1.59) |
|  | secondary | 0.60 (0.51,0.70) | 0.81 (0.65,1.00) | 0.58 (0.34,0.98) | 1.11 (0.48,2.57) |
|  | BSc/MSc | 0.36 (0.15,0.89) | 0.40 (0.10,1.62) | 0.11 (0.02,0.83) | 12.94 (1.16,143.88) |
|  | missing | 0.83 (0.20,3.43) |  | 0.99 (0.30,3.30) |  |
|  |  |  |  |  |  |
| illiterate | no | 1 | 1 | 1 | 1 |
|  | yes | 1.50 (1.32,1.71) | 1.20 (0.98,1.46) | 1.27 (0.74,2.18) | 1.07 (0.68,1.67) |
|  | missing | 2.45 (0.32,18.79) |  | 1.57 (0.48,5.09) |  |
|  |  |  |  |  |  |
| household wealth | 1 | 1.48 (1.27,1.74) | 1.41 (1.11,1.80) | 2.08 (1.07,4.06) | 1.46 (0.70,3.06) |
|  | 2 | 1.22 (1.04,1.43) | 1.22 (1.00,1.49) | 1.20 (0.63,2.30) | 1.19 (0.55,2.57) |
|  | 3 | 1 | 1 | 1 | 1 |
|  | missing |  |  |  |  |
|  |  |  |  |  |  |
| 1 ANC visit | no | 1.19 (1.05,1.35) | 1.21 (1.00,1.46) | 1.38 (0.82,2.33) | 0.93 (0.58,1.50) |
|  | yes | 1 | 1 | 1 | 1 |
|  | missing |  |  |  |  |
|  |  |  |  |  |  |
| 4+ ANC visits | no | 1.18 (0.98,1.41) | 1.29 (1.03,1.61) | 2.16 (1.32,3.53) | 0.86 (0.37,1.98) |
|  | yes | 1 | 1 | 1 | 1 |
|  | missing |  |  |  |  |
|  |  |  |  |  |  |
|  |  |  |  |  |  |
| tetanus vaccination | no | 1.08 (0.96,1.23) | 1.36 (1.09,1.69) | 3.84 (2.08,7.12) | 0.89 (0.40,1.95) |
|  | yes | 1 | 1 | 1 | 1 |
|  | missing |  |  |  | 0.84 (0.55,1.27) |
|  |  |  |  |  |  |
| premature | no | 1 | 1 | 1 | 1 |
|  | yes | 12.04 (10.46,13.84) | 8.54 (6.80,10.74) | 10.92 (5.76,20.71) | 27.09 (16.62,44.15) |
|  | missing | 1.64 (0.77,3.50) | 1.63 (0.71,3.78) |  |  |
|  |  |  |  |  |  |
| pregnancy complications | no | 1 | 1 |  |  |
|  | yes | 1.96 (1.69,2.26) | 1.41 (1.15,1.71) |  |  |
|  | missing | 1.03 (0.14,7.55) |  |  |  |
|  |  |  |  |  |  |
| season | warm | 1 | 1 | 1 | 1 |
|  | rainy | 1.08 (0.92,1.27) | 1.07 (0.85,1.34) | 1.08 (0.55,2.11) | 0.95 (0.59,1.51) |
|  | cold | 1.15 (0.98,1.36) | 1.49 (1.20,1.84) | 1.17 (0.63,2.19) | 1.84 (1.20,2.82) |
|  |  |  |  |  |  |
| delivery location | home | 1 | 1 | 1 | 1 |
|  | institutional | 1.24 (1.08,1.44) | 0.83 (0.65,1.04) | 0.40 (0.23,0.70) | 1.89 (0.67,5.31) |
|  | missing | 1.08 (0.53,2.20) |  |  |  |
|  |  |  |  |  |  |
| labour duration > 24h? | no | 1 | 1 | 1 | 1 |
|  | yes | 1.40 (1.21,1.61) | 1.66 (1.34,2.07) | 1.27 (0.31,5.22) | 2.10 (1.42,3.12) |
|  | missing | 2.15 (0.28,16.40) |  |  |  |
|  |  |  |  |  |  |
| delivery complications | no | 1 | 1 | 1 | 1 |
|  | yes | 2.45 (2.05,2.92) | 1.52 (1.24,1.88) | 5.43 (2.14,13.77) | 1.68 (1.16,2.44) |
|  | missing | 1.01 (0.32,3.20) |  |  |  |
|  |  |  |  |  |  |
| presentation | no | 4.48 (3.48,5.76) | 7.32 (4.69,11.43) |  | 5.34 (1.52,18.71) |
|  | yes | 1 | 1 |  | 1 |
|  | caesarean | 0.71 (0.56,0.89) | 0.76 (0.31,1.86) |  |  |
|  | missing | 2.61 (1.58,4.31) | 1.77 (0.89,3.54) |  | 1.78 (0.23,13.59) |
|  |  |  |  |  |  |
| mother died | no | 1 | 1 | 1 | 1 |
|  | yes | 4.57 (2.05,10.18) | 3.70 (1.71,8.03) | 58.83 (5.27,657.11) | 11.96 (3.63,39.40) |
|  |  |  |  |  |  |
| sex baby | male | 1.37 (1.21,1.55) | 1.39 (1.16,1.66) | 1.37 (0.82, 2.27) | 1.57 (1.07,2.29) |
|  | female | 1 | 1 | 1 | 1 |
|  | missing |  |  |  |  |
|  |  |  |  |  |  |
| multiple birth | no | 1 | 1 | 1 | 1 |
|  | yes | 12.51 (10.28,15.22) | 9.29 (6.85,12.61) | 6.34 (2.68,14.98) | 4.90 (2.35,10.20) |
|  |  |  |  |  |  |
| size at birth | small | 3.34 (2.92,3.82) | 6.75 (5.45,8.36) | 28.04 (12.52,62.81) | 18.36 (11.73,28.74) |
|  | normal | 1 | 1 | 1 | 1 |
|  | large | 2.38 (1.97,2.89) | 2.32 (0.99,5.42) | 4.23 (1.72,10.40) | 5.40 (2.37,12.28) |
|  | missing |  |  | 22.36 (4.54,110.06) |  |
|  |  |  |  |  |  |
| looking abnormal | no | 1 | 1 | 1 | 1 |
|  | yes | 5.90 (5.05,6.89) | 4.69 (3.60,6.10) | 43.76 (21.10,90.74) | 15.75 (9.27,26.73) |
|  | missing | 1.15 (0.97,1.35) | 9.14 (0.83,100.95) |  |  |
|  |  |  |  |  |  |
| breathed & cried | no | 4.49 (3.94,5.11) | 3.93 (1.81,8.55) | 32.99 (18.55,58.65) |  |
| immediately | yes | 1 | 1 | 1 |  |
|  | missing |  |  |  |  |
|  |  |  |  |  |  |
| condition | poor | 11.80 (10.29,13.54) | 24.57 (19.00,31.78) |  |  |
| at 5 mins. | good | 1 | 1 |  |  |
|  | missing | 4.21 (2.90,6.10) | 6.95 (4.26,11.32) |  |  |
|  |  |  |  |  |  |
| condition arms & legs | normal | 1 | 1 |  |  |
|  | floppy | 10.71 (7.61,15.08) | 32.57 (21.92,48.41) |  |  |
|  | stiff | 9.98 (4.85,20.55) | 8.91 (4.01,19.81) |  |  |
|  | missing | 0.93 (0.79,1.09) |  |  |  |

1. The somewhat higher risk of death among facility births compared with home deliveries in Bangladesh is arguably due to selection effects, with high-risk deliveries being more likely to take place in a facility.^29^
2. Residual confounding by socioeconomic position perhaps explains the observed lower risk of death among C-section deliveries compared with vaginal deliveries, especially in the light of the very high C-section rate in Bangladesh (private facilities: 73% of deliveries via C-section; public facilities: 29%),^30^ the strong association between socioeconomic position and C-section delivery in that context,^30^ and the risks associated with elective C-section.^31^

**Supplementary Table 2. Multivariable Associations between Neonatal Mortality and Risk Factors at Start of Pregnancy, Start of Delivery, and Five Minutes after Birth** ΔAIC is reported behind the predictors in bold font; odd ratios (95% confidence intervals) are reported behind predictor levels in regular font.

|  |  | **Start pregnancy** | | **Start delivery** | | **After birth** | |  | **Start delivery (incl multiple birth)** | |
| --- | --- | --- | --- | --- | --- | --- | --- | --- | --- | --- |
|  |  | **All factors** | **Selected factors** | **All factors** | **Selected factors** | **All factors** | **Selected factors** |  | **All factors** | **Selected factors** |
| **site** |  | **189** | **198** | **150** | **166** | **144** | **147** |  | **157** | **174** |
|  | Bangladesh | 1 | 1 | 1 | 1 | 1 | 1 |  | 1 | 1 |
|  | Jharkhand/Odisha, India | 1.57 (1.36,1.80) | 1.66 (1.46,1.89) | 1.56 (1.35,1.82) | 1.61 (1.40,1.84) | 2.21 (1.86,2.62) | 2.04 (1.76,2.37) |  | 1.60 (1.38,1.86) | 1.62 (1.41,1.86) |
|  | Mumbai, India | 0.25 (0.19,0.33) | 0.27 (0.20,0.34) | 0.27 (0.20,0.36) | 0.27 (0.21,0.35) | 0.40 (0.29,0.55) | 0.37 (0.28,0.50) |  | 0.26 (0.19,0.35) | 0.25 (0.19,0.33) |
|  | Nepal | 0.78 (0.62,0.97) | 0.81 (0.65,1.00) | 0.93 (0.73,1.18) | 0.95 (0.76,1.18) | 1.19 (0.91,1.55) | 1.27 (1.00,1.61) |  | 0.94 (0.74,1.19) | 0.97 (0.78,1.21) |
| **time (years)** |  | **-5** |  | **-1** |  | **-8** |  |  | **-2** |  |
|  | 1 | 1 |  | 1 |  | 1 |  |  | 1 |  |
|  | 2 | 1.07 (0.93,1.22) |  | 1.07 (0.93,1.23) |  | 1.03 (0.89,1.20) |  |  | 1.05 (0.91,1.21) |  |
|  | 3 | 1.05 (0.91,1.21) |  | 1.13 (0.97,1.32) |  | 1.06 (0.90,1.25) |  |  | 1.11 (0.95,1.30) |  |
|  | 4 | 0.96 (0.78,1.17) |  | 0.98 (0.79,1.21) |  | 1.16 (0.92,1.46) |  |  | 1.01 (0.82,1.24) |  |
|  | 5 | 0.93 (0.75,1.15) |  | 0.88 (0.70,1.10) |  | 1.15 (0.90,1.47) |  |  | 0.88 (0.70,1.10) |  |
|  | 6 | 0.87 (0.72,1.06) |  | 0.88 (0.72,1.08) |  | 1.10 (0.88,1.38) |  |  | 0.87 (0.71,1.08) |  |
| **age** |  | **14** | **14** | **1** |  | **-3** |  |  | **3** |  |
|  | <18 | 1.56 (1.26,1.93) | 1.55 (1.25,1.92) | 1.25 (1.00,1.55) |  | 1.11 (0.87,1.41) |  |  | 1.31 (1.05,1.64) |  |
|  | 18-20 | 1.30 (1.14,1.48) | 1.30 (1.14,1.48) | 1.14 (0.99,1.30) |  | 1.06 (0.92,1.23) |  |  | 1.17 (1.02,1.34) |  |
|  | 21-23 | 1.11 (1.05,1.17) | 1.11 (1.05,1.17) | 1.05 (0.99,1.11) |  | 1.02 (0.96,1.09) |  |  | 1.06 (1.00,1.12) |  |
|  | 24-26 | 1 | 1 | 1 |  | 1 |  |  | 1 |  |
|  | 27-29 | 0.99 (0.96,1.03) | 0.99 (0.96,1.03) | 1.01 (0.97,1.04) |  | 1.01 (0.97,1.04) |  |  | 1.00 (0.97,1.04) |  |
|  | 30-32 | 1.04 (0.97,1.12) | 1.05 (0.98,1.12) | 1.04 (0.97,1.12) |  | 1.03 (0.95,1.12) |  |  | 1.04 (0.97,1.12) |  |
|  | 33-35 | 1.14 (1.00,1.29) | 1.14 (1.00,1.29) | 1.11 (0.97,1.26) |  | 1.07 (0.93,1.23) |  |  | 1.11 (0.97,1.27) |  |
|  | >35 | 1.26 (1.03,1.54) | 1.27 (1.04,1.54) | 1.19 (0.96,1.46) |  | 1.12 (0.90,1.40) |  |  | 1.20 (0.97,1.49) |  |
| **birth interval (months)** | | **31** | **31** | **22** | **41** | **15** | **20** |  | **27** | **55** |
|  | primi gravida | 1.34 (1.14,1.57) | 1.34 (1.14,1.57) | 1.33 (1.12,1.57) | 1.40 (1.22,1.60) | 1.22 (1.02,1.46) | 1.25 (1.08,1.44) |  | 1.41 (1.19,1.67) | 1.50 (1.31,1.72) |
|  | <15 | 2.18 (1.69,2.82) | 2.18 (1.69,2.82) | 1.88 (1.44,2.43) | 1.90 (1.48,2.45) | 1.85 (1.39,2.45) | 1.82 (1.38,2.40) |  | 1.95 (1.49,2.56) | 1.98 (1.53,2.58) |
|  | 15-26 | 1.11 (0.92,1.33) | 1.11 (0.93,1.33) | 1.00 (0.82,1.21) | 1.01 (0.83,1.22) | 0.99 (0.81,1.22) | 1.01 (0.82,1.23) |  | 1.02 (0.84,1.25) | 1.04 (0.85,1.26) |
|  | 27-68 | 1 | 1 | 1 | 1 | 1 | 1 |  | 1 | 1 |
|  | >68 | 1.17 (0.96,1.42) | 1.16 (0.95,1.41) | 1.08 (0.88,1.32) | 1.07 (0.88,1.30) | 1.00 (0.81,1.24) | 1.02 (0.83,1.25) |  | 1.05 (0.86,1.29) | 1.04 (0.85,1.26) |
| **education** |  | **8** | **35** | **15** | **50** | **7** | **41** |  | **17** | **49** |
|  | no school | 1 | 1 | 1 | 1 | 1 | 1 |  | 1 | 1 |
|  | primary | 0.91 (0.72,1.14) | 0.84 (0.73,0.97) | 0.79 (0.62,1.00) | 0.79 (0.68,0.91) | 0.82 (0.63,1.06) | 0.80 (0.69,0.94) |  | 0.79 (0.62,1.01) | 0.80 (0.69,0.93) |
|  | secondary | 0.72 (0.56,0.93) | 0.65 (0.57,0.75) | 0.61 (0.47,0.80) | 0.60 (0.52,0.69) | 0.65 (0.48,0.87) | 0.62 (0.53,0.71) |  | 0.61 (0.46,0.80) | 0.61 (0.53,0.70) |
|  | BSc/MSc | 0.43 (0.21,0.86) | 0.39 (0.20,0.76) | 0.32 (0.16,0.66) | 0.30 (0.15,0.59) | 0.40 (0.19,0.86) | 0.37 (0.18,0.75) |  | 0.27 (0.13,0.56) | 0.25 (0.12,0.50) |
| **illiterate** |  | **-1** |  | **-2** |  | **-2** |  |  | **-2** |  |
|  | no | 1 |  | 1 |  | 1 |  |  | 1 |  |
|  | yes | 1.09 (0.87,1.37) |  | 0.98 (0.77,1.24) |  | 0.97 (0.75,1.26) |  |  | 0.96 (0.75,1.21) |  |
| **household wealth (tertiles)** | | **10** | **12** | **9** | **12** | **2** |  |  | **9** | **13** |
|  | 1 | 1.29 (1.13,1.48) | 1.31 (1.14,1.50) | 1.29 (1.12,1.49) | 1.32 (1.15,1.52) | 1.20 (1.03,1.40) |  |  | 1.29 (1.12,1.49) | 1.33 (1.16,1.53) |
|  | 2 | 1.14 (1.00,1.29) | 1.12 (0.99,1.27) | 1.10 (0.96,1.25) | 1.10 (0.97,1.25) | 1.05 (0.91,1.21) |  |  | 1.09 (0.96,1.25) | 1.11 (0.97,1.26) |
|  | 3 | 1 | 1 | 1 | 1 | 1 |  |  | 1 | 1 |
| **1 ANC visit** |  |  |  | **-2** |  | **1** |  |  | **-1** |  |
|  | no |  |  | 1 |  | 1 |  |  | 1 |  |
|  | yes |  |  | 0.98 (0.87,1.10) |  | 0.90 (0.79,1.02) |  |  | 0.93 (0.83,1.05) |  |
| **4+ ANC visits** |  |  |  | **-2** |  | **-2** |  |  | **-2** |  |
|  | no |  |  | 1 |  | 1 |  |  | 1 |  |
|  | yes |  |  | 0.97 (0.83,1.13) |  | 0.98 (0.83,1.16) |  |  | 0.95 (0.82,1.12) |  |
| **tetanus vaccination** | |  |  | **2** |  | **-2** |  |  | **1** |  |
|  | no |  |  | 1 |  | 1 |  |  | 1 |  |
|  | yes |  |  | 0.89 (0.79,1.00) |  | 0.97 (0.86,1.11) |  |  | 0.90 (0.79,1.01) |  |
| **premature** |  |  |  | **1621** | **1658** | **711** | **745** |  | **1335** | **1372** |
|  | no |  |  | 1 | 1 | 1 | 1 |  | 1 | 1 |
|  | yes |  |  | 11.01 (9.80,12.38) | 11.11 (9.89,12.47) | 7.44 (6.42,8.62) | 7.62 (6.59,8.82) |  | 9.52 (8.44,10.75) | 9.65 (8.56,10.88) |
| **pregnancy complications** | |  |  | **46** | **46** | **18** | **22** |  | **41** | **40** |
|  | no |  |  | 1 | 1 | 1 | 1 |  | 1 | 1 |
|  | yes |  |  | 1.55 (1.37,1.75) | 1.55 (1.37,1.75) | 1.35 (1.18,1.54) | 1.40 (1.22,1.59) |  | 1.52 (1.34,1.72) | 1.51 (1.33,1.71) |
| **season** |  |  |  | **12** | **13** | **22** | **23** |  | **13** | **15** |
|  | warm |  |  | 1 | 1 | 1 | 1 |  | 1 | 1 |
|  | rainy |  |  | 0.99 (0.87,1.13) | 1.00 (0.88,1.14) | 1.06 (0.92,1.22) | 1.05 (0.91,1.21) |  | 1.00 (0.88,1.14) | 1.01 (0.89,1.16) |
|  | cold |  |  | 1.23 (1.08,1.39) | 1.24 (1.09,1.41) | 1.38 (1.20,1.59) | 1.38 (1.20,1.59) |  | 1.25 (1.10,1.42) | 1.27 (1.11,1.44) |
| **delivery location** | |  |  |  |  | **1** |  |  |  |  |
|  | home |  |  |  |  | 1 |  |  |  |  |
|  | institutional |  |  |  |  | 1.16 (0.98,1.36) |  |  |  |  |
| **labour duration > 24h?** | |  |  |  |  | **-1** |  |  |  |  |
|  | no |  |  |  |  | 1 |  |  |  |  |
|  | yes |  |  |  |  | 1.06 (0.92,1.21) |  |  |  |  |
| **Delivery problems** | |  |  |  |  | **6** |  |  |  |  |
|  | no |  |  |  |  | 1 |  |  |  |  |
|  | yes |  |  |  |  | 1.25 (1.07,1.45) |  |  |  |  |
| **presentation** |  |  |  |  |  | **47** | **49** |  |  |  |
|  | caesarean |  |  |  |  | 0.45 (0.34,0.59) | 0.47 (0.36,0.60) |  |  |  |
|  | breech |  |  |  |  | 1.75 (1.32,2.31) | 1.75 (1.33,2.32) |  |  |  |
|  | normal |  |  |  |  | 1 | 1 |  |  |  |
| **mother died** |  |  |  |  |  | **3** |  |  |  |  |
|  | no |  |  |  |  | 1 |  |  |  |  |
|  | yes |  |  |  |  | 2.08 (1.09,3.97) |  |  |  |  |
| **sex baby** |  |  |  |  |  | **30** | **31** |  |  |  |
|  | male |  |  |  |  | 1.38 (1.23,1.54) | 1.38 (1.24,1.54) |  |  |  |
|  | female |  |  |  |  | 1 | 1 |  |  |  |
| **multiple birth** |  |  |  |  |  | **329** | **333** |  | **511** | **508** |
|  | no |  |  |  |  | 1 | 1 |  | 1 | 1 |
|  | yes |  |  |  |  | 6.75 (5.49,8.29) | 6.78 (5.52,8.32) |  | 7.76 (6.50,9.27) | 7.67 (6.43,9.16) |
| **size at birth** |  |  |  |  |  | **76** | **82** |  |  |  |
|  | small |  |  |  |  | 1.47 (1.27,1.70) | 1.50 (1.31,1.73) |  |  |  |
|  | normal |  |  |  |  | 1 | 1 |  |  |  |
|  | large |  |  |  |  | 2.25 (1.86,2.73) | 2.29 (1.89,2.77) |  |  |  |
| **looking abnormal** | |  |  |  |  | **-2** |  |  |  |  |
|  | no |  |  |  |  | 1 |  |  |  |  |
|  | yes |  |  |  |  | 1.04 (0.85,1.26) |  |  |  |  |
| **condition at 5m** | |  |  |  |  | **876** | **1110** |  |  |  |
|  | poor |  |  |  |  | 9.70 (8.34,11.27) | 10.09 (8.81,11.56) |  |  |  |
|  | good |  |  |  |  | 1 | 1 |  |  |  |
| **condition arms** | |  |  |  |  | **111** | **119** |  |  |  |
|  | normal |  |  |  |  | 1 | 1 |  |  |  |
|  | floppy |  |  |  |  | 5.16 (3.82,6.97) | 5.25 (3.91,7.05) |  |  |  |
|  | stiff |  |  |  |  | 2.25 (1.09,4.61) | 2.29 (1.13,4.65) |  |  |  |
| **AUC apparent validation** | |  |  |  |  |  |  |  |  |  |
| Rural Bangladesh | | 0.60 (0.58,0.61) | 0.59 (0.58,0.61) | 0.73 (0.71,0.75) | 0.73 (0.71,0.75) | 0.83 (0.82,0.85) | 0.83 (0.81,0.84) |  | 0.75 (0.74,0.77) | 0.75 (0.74,0.77) |
| Rural India |  | 0.60 (0.57,0.63) | 0.60 (0.57,0.62) | 0.69 (0.66,0.71) | 0.68 (0.65,0.71) | 0.80 (0.78,0.83) | 0.80 (0.78,0.82) |  | 0.71 (0.69,0.74) | 0.71 (0.68,0.73) |
| Urban India |  | 0.63 (0.55,0.70) | 0.63 (0.56,0.70) | 0.76 (0.69,0.83) | 0.75 (0.68,0.83) | 0.92 (0.89,0.96) | 0.92 (0.88,0.96) |  | 0.76 (0.69,0.83) | 0.75 (0.68,0.82) |
| Rural Nepal |  | 0.53 (0.46,0.60) | 0.54 (0.47,0.61) | 0.71 (0.65,0.77) | 0.71 (0.66,0.77) | 0.85 (0.80,0.90) | 0.84 (0.79,0.89) |  | 0.72 (0.66,0.78) | 0.73 (0.67,0.79) |
| Pooled average |  | 0.59 (0.58,0.61) | 0.59 (0.58,0.61) | 0.72 (0.69,0.75) | 0.72 (0.68,0.75) | 0.85 (0.81,0.89) | 0.85 (0.80,0.89) |  | 0.73 (0.71,0.76) | 0.73 (0.70,0.76) |
| **AUC cross validation** | |  |  |  |  |  |  |  |  |  |
| Rural Bangladesh | | 0.58 (0.56,0.59) | 0.58 (0.56,0.60) | 0.71 (0.69,0.73) | 0.72 (0.70,0.74) | 0.80 (0.79,0.82) | 0.81 (0.79,0.83) |  | 0.74 (0.72,0.75) | 0.74 (0.72,0.76) |
| Rural India |  | 0.58 (0.56,0.61) | 0.58 (0.56,0.61) | 0.68 (0.65,0.70) | 0.67 (0.65,0.70) | 0.79 (0.77,0.82) | 0.79 (0.77,0.82) |  | 0.70 (0.68,0.73) | 0.70 (0.67,0.73) |
| Urban India |  | 0.61 (0.53,0.68) | 0.62 (0.55,0.69) | 0.76 (0.68,0.83) | 0.75 (0.68,0.83) | 0.90 (0.86,0.95) | 0.90 (0.85,0.95) |  | 0.75 (0.68,0.82) | 0.75 (0.67,0.82) |
| Rural Nepal |  | 0.53 (0.47,0.60) | 0.53 (0.47,0.60) | 0.70 (0.64,0.76) | 0.71 (0.65,0.77) | 0.84 (0.79,0.89) | 0.84 (0.79,0.89) |  | 0.71 (0.65,0.77) | 0.72 (0.66,0.78) |
| Pooled average |  | 0.58 (0.56,0.59) | 0.58 (0.56,0.59) | 0.70 (0.68,0.73) | 0.71 (0.68,0.74) | 0.83 (0.79,0.87) | 0.83 (0.79,0.86) |  | 0.72 (0.70,0.74) | 0.73 (0.70,0.75) |

**Supplementary Table 3. Nomogram details**

To estimate an infant’s probability of neonatal death, first determine all its risk factor characteristics. Second, read the risk points associated with each risk factor from the table. Third, add up the points for all risk factors to obtain the total points for that infant. Based on the model’s intercept and slope (last two rows of the table), the probability of neonatal death can be calculated with the formula: $\frac{1}{1+\text{exp}\left[ -\left[ \text{intercept }+ \text{total points }\times\text{slope} \right] \right]}$ .

|  |  | **Start pregnancy** | **Start delivery** | **Start delivery (incl multiple birth)** | **After birth** |
| --- | --- | --- | --- | --- | --- |
| site | rural Bangladesh | 3.6 | 2.7 | 3.1 | 2.1 |
|  | Jharkhand/Odisha India | 5.0 | 3.7 | 4.1 | 3.7 |
|  | Mumbai, India | 0.0 | 0.0 | 0.0 | 0.0 |
|  | rural Nepal | 3.0 | 2.6 | 3.0 | 2.6 |
|  |  |  |  |  |  |
| age | 15 | 1.5 |  |  |  |
|  | 16 | 1.3 |  |  |  |
|  | 17 | 1.1 |  |  |  |
|  | 18 | 1.0 |  |  |  |
|  | 19 | 0.8 |  |  |  |
|  | 20 | 0.6 |  |  |  |
|  | 21 | 0.5 |  |  |  |
|  | 22 | 0.3 |  |  |  |
|  | 23 | 0.2 |  |  |  |
|  | 24 | 0.1 |  |  |  |
|  | 25 | 0.0 |  |  |  |
|  | 26 | 0.0 |  |  |  |
|  | 27 | 0.0 |  |  |  |
|  | 28 | 0.0 |  |  |  |
|  | 29 | 0.1 |  |  |  |
|  | 30 | 0.1 |  |  |  |
|  | 31 | 0.2 |  |  |  |
|  | 32 | 0.2 |  |  |  |
|  | 33 | 0.3 |  |  |  |
|  | 34 | 0.4 |  |  |  |
|  | 35 | 0.4 |  |  |  |
|  | 36 | 0.5 |  |  |  |
|  | 37 | 0.6 |  |  |  |
|  | 38 | 0.6 |  |  |  |
|  | 39 | 0.7 |  |  |  |
|  | 40 | 0.8 |  |  |  |
|  |  |  |  |  |  |
| birth interval | <15 months | 2.1 | 1.3 | 1.5 | 1.4 |
|  | >=15 months | 0.0 | 0.0 | 0.0 | 0.0 |
|  | primi | 0.8 | 0.7 | 0.9 | 0.5 |
|  |  |  |  |  |  |
| education | no school | 2.6 | 2.5 | 3.1 | 2.2 |
|  | primary | 2.1 | 2.0 | 2.6 | 1.7 |
|  | secondary | 1.4 | 1.5 | 2.0 | 1.1 |
|  | BSc/MSc | 0.0 | 0.0 | 0.0 | 0.0 |
|  |  |  |  |  |  |
|  |  |  |  |  |  |
| household wealth (tertiles) | poorest | 0.7 | 0.6 | 0.6 |  |
|  | middle | 0.3 | 0.2 | 0.2 |  |
|  | least-poor | 0.0 | 0.0 | 0.0 |  |
|  |  |  |  |  |  |
| premature | no |  | 0.0 | 0.0 | 0.0 |
|  | yes |  | 5.0 | 5.0 | 4.4 |
|  |  |  |  |  |  |
| pregnancy complications | no |  | 0.0 | 0.0 | 0.0 |
|  | yes |  | 0.9 | 0.9 | 0.7 |
|  |  |  |  |  |  |
| season | warm |  | 0.0 | 0.0 | 0.0 |
|  | rainy |  | 0.0 | 0.0 | 0.1 |
|  | cold |  | 0.5 | 0.5 | 0.7 |
|  |  |  |  |  |  |
| multiple birth | singleton |  |  | 0.0 | 0.0 |
|  | twin/multiple |  |  | 4.5 | 4.2 |
|  |  |  |  |  |  |
| presentation | normal |  |  |  | 1.7 |
|  | caesarian |  |  |  | 0.0 |
|  | breech |  |  |  | 2.9 |
|  |  |  |  |  |  |
| sex baby | male |  |  |  | 0.7 |
|  | female |  |  |  | 0.0 |
|  |  |  |  |  |  |
| size at birth | small |  |  |  | 0.9 |
|  | normal |  |  |  | 0.0 |
|  | large |  |  |  | 1.8 |
|  |  |  |  |  |  |
| good condition at 5 min | no |  |  |  | 5.0 |
|  | yes |  |  |  | 0.0 |
|  |  |  |  |  |  |
| condition arms/legs | normal |  |  |  | 0.0 |
|  | floppy |  |  |  | 3.6 |
|  | stiff |  |  |  | 1.8 |
|  |  |  |  |  |  |
|  | intercept | -5.8191 | -6.3908 | -6.7802 | -7.3141 |
|  | slope | 0.3675 | 0.4815 | 0.4534 | 0.4624 |
